# Determination of Dicofol in Tea Using Surface-Enhanced Raman Spectroscopy Coupled Chemometrics

**DOI:** 10.3390/molecules28145291

**Published:** 2023-07-08

**Authors:** Qian Ke, Limei Yin, Heera Jayan, Hesham R. El-Seedi, Paula L. Gómez, Stella M. Alzamora, Xiaobo Zou, Zhiming Guo

**Affiliations:** 1 School of Food and Biological Engineering, Jiangsu University, Zhenjiang 212013, China; keqian2021@163.com (Q.K.); yinlm6@163.com (L.Y.); heerajayan93@outlook.com (H.J.); 2 China Light Industry Key Laboratory of Food Intelligent Detection & Processing, Jiangsu University, Zhenjiang 212013, China; zou_xiaobo@ujs.edu.cn; 3 Pharmacognosy Group, Department of Pharmaceutical Biosciences, BMC, Uppsala University, P.O. Box 591, SE 751 24 Uppsala, Sweden; hesham.el-seedi@fkog.uu.se; 4 International Research Center for Food Nutrition and Safety, Jiangsu University, Zhenjiang 212013, China; 5 Consejo Nacional de Investigaciones Cientificasy Tecnicas (CONICET), University of Buenos Aires, Ciudad Autónoma de Buenos Aires C1428EGA, Argentina; gzpaula@gmail.com (P.L.G.); smalzamora@gmail.com (S.M.A.); 6 International Joint Research Laboratory of Intelligent Agriculture and Agri-Products Processing, Jiangsu University, Zhenjiang 212013, China

**Keywords:** dicofol, tea, SERS, Au@AgNPs/PDMS, chemometrics

## Abstract

Dicofol is a highly toxic residual pesticide in tea, which seriously endangers human health. A method for detecting dicofol in tea by combining stoichiometry with surface-enhanced Raman spectroscopy (SERS) technology was proposed in this study. AuNPs were prepared, and silver shells were grown on the surface of AuNPs to obtain core–shell Au@AgNPs. Then, the core–shell Au@AgNPs were attached to the surface of a PDMS membrane by physical deposition to obtain a Au@AgNPs/PDMS substrate. The limit of detection (LOD) of this substrate for 4-ATP is as low as 0.28 × 10^−11^ mol/L, and the LOD of dicofol in tea is 0.32 ng/kg, showing high sensitivity. By comparing the modeling effects of preprocessing and variable selection algorithms, it is concluded that the modeling effect of Savitzky–Golay combined with competitive adaptive reweighted sampling–partial least squares regression is the best (Rp = 0.9964, RPD = 10.6145). SERS technology combined with stoichiometry is expected to rapidly detect dicofol in tea without labels.

## 1. Introduction

Tea is the second-most-popular drink around the world after water and is known for its unique aroma and texture [1,2]. As the birthplace of tea, China has a rich tea culture and is one of the world’s largest tea producers and major exporters [3]. Tea is diverse and contains a variety of organic compounds and inorganic mineral elements [4]. Studies showed that regularly drinking tea can combat several chronic devastating disorders such as cancer, diabetes, cardiovascular diseases, and neurological diseases [5,6].

During the growth of tea, people use different kinds of pesticides to fight pests and diseases. Organochlorine pesticides are the most widely used class of synthetic organic pesticides so far [7]. However, this type of pesticide is chemically stable and has a long residue time in the environment, causing serious environmental pollution [8,9]. Dicofol is a highly effective organochlorine pesticide that has been widely used in agricultural pest control [10]. However, it is not easy to degrade under natural conditions. It is also known to be toxic and harmful, can cause human liver dysfunction and carcinogenic symptoms, and has long been banned in China. However, as the residue period increases, pesticides have a cumulative effect in the soil and are sometimes detected in certain water quality and soil samples. Dicofol is still detected in some teas as pesticide residues in water and soil can be immersed in tea leaves along with tea plants, endangering human health. GB 2763-2021 stipulates that the maximum residue limit of dicofol in tea is 0.2 mg/kg. The highly efficient and sensitive detection of dicofol in tea reduces the risk of contaminated tea entering the production and circulation links from the source and has certain social and economic application value.

The detection and analysis methods of organochlorine pesticides mainly include liquid chromatography and gas chromatography [11,12]. Although these methods have high sensitivity and good reproducibility, the preprocessing steps are complex, the detection efficiency is low, and the detection cost is high [13]. In addition, the requirements for operators are high, so it is not suitable for the detection of large-scale experimental samples [14,15]. Therefore, it is necessary to establish an efficient, economical, and sensitive detection method to detect dicofol in tea.

Raman scattering is caused by the vibration of functional groups within molecules, and different functional groups produce different characteristic vibration energy levels; thus, each molecule has its own unique Raman spectrum, which we call the fingerprint characteristics of Raman spectroscopy [16]. However, the signal intensity of the Raman spectrum is weak (only 10^−6^ of incident light), which is difficult to observe. The discovery of the surface-enhanced Raman scattering (SERS) effect made the application of Raman spectroscopy possible. SERS technique relies on the local electromagnetic enhancement caused by the plasmon resonance on a rough metal surface or the charge transfer between metal nanomaterials and target analytes to amplify the Raman signal of the target analytes. [17,18,19]. SERS technology has a wide range of applications in medicine, food safety, and the life sciences [20,21,22].

The preparation of high-performance SERS substrates is particularly critical to the development of SERS technology [23,24]. The precious metals gold and silver are commonly used as enhanced substrates in SERS detection and analysis [25]. Gold nanoparticles have high stability, while silver nanoparticles have high sensitivity, and their morphology and size are controllable, have good reproducibility, and can be uniformly adsorbed on target molecules, with superior surface-enhanced Raman properties. The gold–silver core–shell nanostructure integrates the advantages of the two precious metals, the stability is good, and the sensitivity is further improved; thus, the application range is wider [26]. PDMS film is a commonly used flexible material for supported metal nanoparticles, with good flexibility and tensile properties, strong corrosion resistance, and good stability, and is an ideal substrate material [27,28,29].

**Figure 1:**
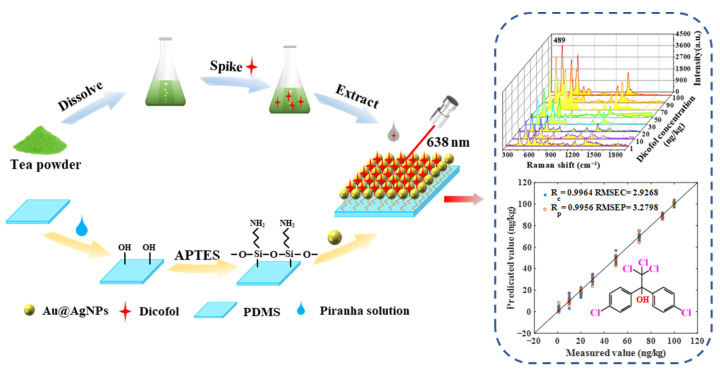
Schematic diagram of Au@AgNPs/PDMS as a surface-enhanced Raman scattering substrate for the determination of dicofol in tea.

This study proposed a method for the rapid and highly sensitive detection of dicofol in tea by combining core–shell Au@AgNPs and PDMS film (Figure 1). After a flexible PDMS membrane is immersed in 3-aminopropyltriethoxysilane (APTES) solution, the surface is modified with a certain quantity of amino acids, which can better bind to core–shell Au@AgNPs [30,31]. At an excitation wavelength of 638 nm, the Raman spectra of the gradient concentration of the tea samples added with dicofol standard substance on a Au@AgNPs/PDMS substrate were acquired, and a linear relationship was established. The results showed that in the linear range of 10–100 ng/kg, there was a strong correlation between the concentration of dicofol and the SERS signal intensity. To eliminate the influence of noise interference and further improve the correlation, the original spectrum is preprocessed, and the variable selection algorithm is used. The best combination of models for the quantitative detection of dicofol in tea was determined by comparing Rp and RSD. SERS technology combined with stoichiometry is expected to rapidly detect dicofol in tea without labels.

## 2. Results

### 2.1. Optimization of AuNPs and Au@AgNPs

The enhancement of the SERS signal is mainly caused by the local electric field enhancement of the plasma on the metal surface. Therefore, the controllability and uniformity of the particle size and distribution in nanoparticle systems is the key to constructing SERS substrates with strong stability, high sensitivity, and a uniform signal [32]. This study prepared AuNPs with different particle sizes and grew silver shells with different thicknesses on the surface of AuNPs to obtain core–shell Au@AgNPs. Figure S1a–f is TEM characterizations of AuNPs prepared by adding different volumes (150, 200, 250, 300, 350, and 400 μL) of trisodium citrate to HAuCl_4_ solution. It can be observed that when 150 and 200 μL of trisodium citrate are added, the synthesized AuNPs are uneven in size, with particle sizes of 80 nm and 50 nm, respectively. When the volume of trisodium citrate increased from 250 μL to 300, 350, and 400 μL, the shape of the AuNPs gradually approached a spherical shape, and the particle size decreased from 34 nm to 25, 23, and 19 nm, respectively.

The thickness of the silver shell is determined by the volumes of the AgNO_3_ and ascorbic acid. Figure S1g–l shows the TEM images of core–shell Au@AgNPs prepared by dropping different amounts of AgNO_3_ and ascorbic acid in AuNPs (34 nm) solution. It can be observed that the core–shell Au@AgNPs’ center is darker, and the edges are brightly colored, which accords with the characteristics of the core–shell nanostructure [33,34]. The thickness of the Ag shell increased from 3 nm to 4.5, 5.5, 9, 11, and 15 nm when AgNO_3_ and ascorbic acid were added at 30, 45, 60, 75, 90, and 105 μL, respectively.

### 2.2. Characterization of AuNPs and Core–Shell Au@AgNPs

**Figure 2:**
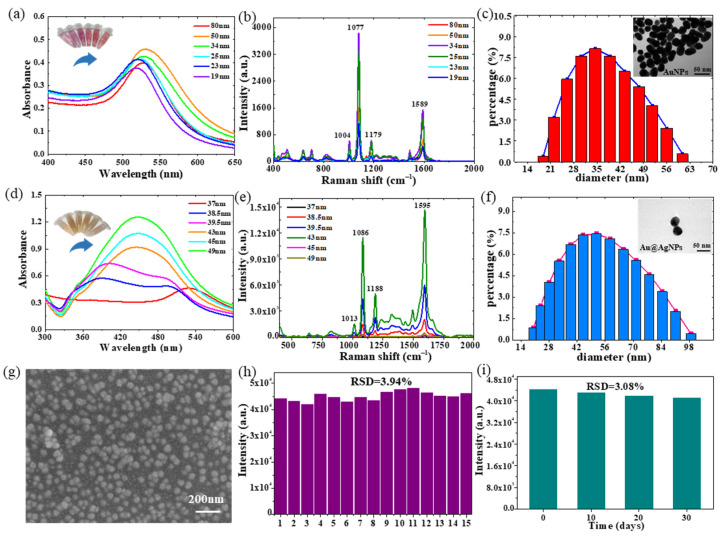
(**a**) UV spectra and (**b**) Raman spectra of AuNPs of different sizes; (**c**) particle size distribution of 34 nm AuNPs; (**d**) UV spectra and (**e**) Raman spectra of core–shell Au@AgNPs with Ag shell thicknesses of different sizes; (**f**) particle size distribution of core–shell Au@AgNPs with Ag shell thickness of 9 nm; (**g**) TEM results of the Au@AgNPs/PDMS substrate; (**h**) SERS intensity of 4-ATP at 1078 cm^−1^ on 3 mm × 2 mm Au@AgNPs/PDMS; (**i**) SERS intensity of 4-ATP (at 1078 cm^−1^) on Au@AgNPs/PDMS substrates stored for 0, 10, 20, and 30 days, respectively.

Trisodium citrate (150, 200, 250, 300, 350, and 400 μL) was added to HAuCl_4_ to prepare AuNPs with different particle sizes. The UV results are shown in Figure 2a. With the increase in sodium citrate, the peak position of the localized surface plasmon resonance (LSPR) moved blue. The LSPR band is located at 535 nm for the 80 nm AuNPs and 523 nm for the 19 nm AuNPs, with a blue shift of 12 nm. The color of the AuNPs solution changed from dark purple to light orange. Figure 2b shows that the Raman intensity of AuNPs gradually increases when the volume of trisodium citrate increases from 150 to 250 μL and then gradually decreases with the increase in the addition amount. AuNPs (34 nm) synthesized with a volume of 250 μL trisodium citrate had the highest Raman intensity. Figure 2c is the histogram of the particle size distribution of the 34 nm AuNPs, which shows that the distribution of the synthesized AuNPs is relatively uniform. The upper-right corner of the histogram shows the TEM image of the particle, which demonstrates that the prepared AuNPs were relatively uniform. Therefore, 34 nm AuNPs were selected for the core–shell Au@AgNPs’ synthesis.

To explore the effect of silver-shell thickness on the core–shell Au@AgNPs’ LSPR characteristics, we collected the UV spectra of the core–shell Au@AgNPs’ solutions with different silver-shell thicknesses, as shown in Figure 2d. There were two peaks in the Au@AgNPs’ Raman spectrum when AgNO_3_ and ascorbic acid were added at 30 μL. The peak at 374 nm is the Ag shell peak, and the peak at 528 nm is the Au nucleus peak. With the thickening of the Ag shell, the LSPR of the Ag shell gradually strengthens, and the LSPR of the Au nucleus gradually weakens or is even completely shielded. It can be seen from Figure 2d that the peak around 528 nm gradually disappears, while the peak around 374 nm gradually becomes obvious and redshifted. When the Ag shell thickness exceeds a certain value (9 nm), the Au nuclear LSPR disappears, leaving only the LSPR of the Ag shell. The core–shell Au@AgNPs’ solution changes from orange to light yellow. Figure 2e shows that when the addition of ascorbic acid and AgNO_3_ increased from 30 to 75 μL, the Raman intensity of the core–shell Au@AgNPs gradually increases and then decreases with the increase in the addition amount. When the amount of ascorbic acid and AgNO_3_ is 75 μL, the thickness of silver shell is 9 nm, and the Raman strength of the core–shell Au@AgNPs is the highest. Figure 2f shows that the size distribution of the core–shell Au@AgNPs’ solution’s particles at this thickness is relatively uniform. The TEM image at the upper right shows that the synthesis of the particles is relatively uniform. Subsequent operations use the size of the core–shell Au@AgNPs.

### 2.3. Characterization of the Au@AgNPs/PDMS Substrate

Before collecting the Raman spectra of the target substance, the Raman spectra of the blank tin foil, blank PDMS, and blank Au@AgNPs/PDMS substrate were collected, and the results are shown in Figure S2a,b. The intensity of the blank tin foil, blank PDMS, and blank Au@AgNPs/PDMS substrates is less than 100, and the influence on the subsequent detection of the target substances is negligible. The TEM results of the Au@AgNPs/PDMS substrate are shown in Figure 2g, where the core–shell Au@AgNPs is spherical and uniformly distributed on the PDMS membrane. The diameter of a single core–shell Au@AgNP is about 45 nm. To evaluate the SERS-enhanced effect of the Au@AgNPs/PDMS substrate, 4-ATP solution (10^−6^ mol/L) was dripped onto the tin foil and SERS-enhanced substrate, and the Raman spectrum was collected (Figure S2b). The band intensity of the 4-ATP located around 1078 cm^−1^ is 278 on the tin foil and 41,702.3 on the reinforced substrate. It can be seen that the Au@AgNPs/PDMS substrate has a good SERS-enhancement effect. In addition, the original Raman spectra of the 4-ATP on the tin foil showed significant Raman peaks at 473, 876, 1087, and 1588cm^−1^. They belong to the C-C out-of-plane coupled vibration (473 cm^−1^), the C-S out-of-plane coupled vibration (876 cm^−1^), the C-S in-plane tensile vibration (1087 cm^−1^), and the C-C in-plane tensile vibration (1588 cm^−1^), respectively [35,36]. When 4-ATP4-ATP is attached to Au@AgNPs/PDMS, a Ag-N bond is formed, the out-of-plane vibration is weakened, and the Raman peaks at 473 cm^−1^ and 876 cm^−1^ disappear [37].

### 2.4. Reproducibility and Stability Analysis

To verify the reproducibility of the Au@AgNPs/PDMS substrate, 5 μL 4-ATP (0.01 mol L^−1^) solution was dropped onto the SERS substrate, and 15 Raman spectra were collected. The relative standard deviation was calculated at the spectral intensity of 1078 cm^−1^. The relative standard deviation (RSD) is 3.94% (shown in Figure 2h), indicating the good reproducibility of the SERS substrate. After storing the Au@AgNPs/PDMS substrate for 10 days, 20 days, and 30 days, 5 μL of 4-ATP (0.01 mol L^−1^) solution was dropped to acquire the Raman spectra. Figure 2i shows that compared with the newly prepared substrate, the Raman intensity of the SERS base (1078 cm^−1^) slightly decreased after 30 days of storage. The results indicated that the substrate stability was excellent (RSD = 3.08%). The results showed that the stability of the Au@AgNPs/PDMS substrate is good and can be stored for at least one month.

### 2.5. Sensitivity of Au@AgNPs/PDMS

**Figure 3:**
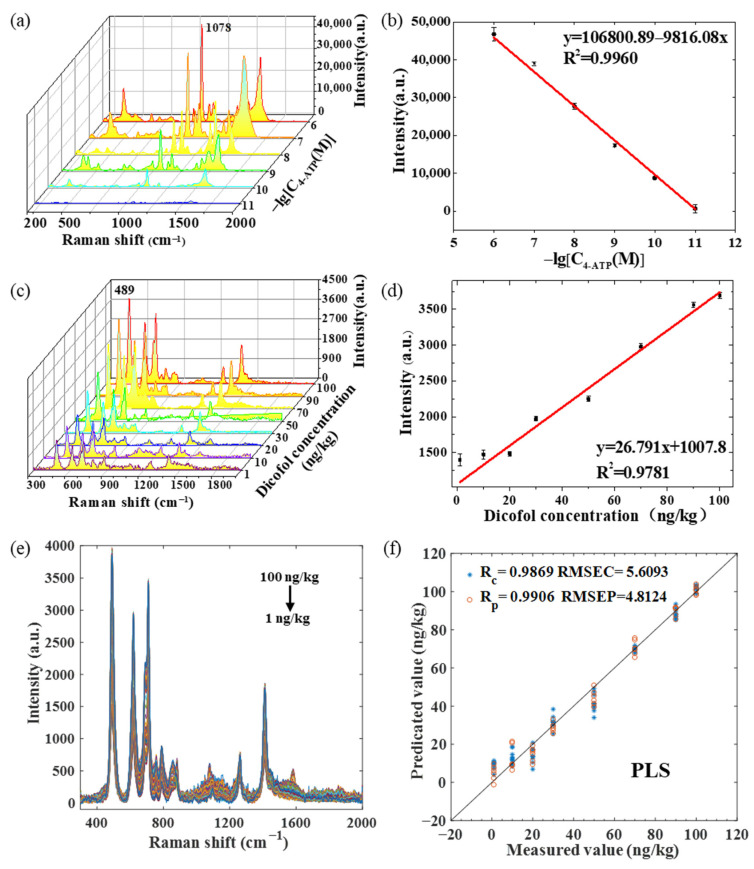
(**a**) Raman spectra of gradient concentration of 4-ATP; (**b**) standard curve of Raman intensity and logarithmic concentration of 4-ATP at 1078 cm^−1^; (**c**) Raman spectra of eight gradient concentrations of dicofol; (**d**) standard curve of Raman intensity and concentration of dicofol at 489 cm^−1^; (**e**) raw Raman spectra of dicofol under eight concentration gradients; (**f**) PLS modeling results of the original Raman spectrum.

To further explore the sensitivity of the Au@AgNPs/PDMS substrates, the Raman spectra of 4-ATP solutions with gradient concentrations of 10^−6^, 10^−7^, 10^−8^, 10^−9^, 10^−10^, and 10^−11^ mol/L were collected. Figure 3a shows that as the 4-ATP concentration decreases, the Raman intensity also decreases, and the SERS signal is still visible when the 4-ATP concentration is as low as 10^−11^ mol/L (the SERS intensity is 822). This indicates that the Au@AgNPs/PDMS substrate has a high sensitivity. Figure 3b shows the relationship between the Raman intensity and logarithmic concentration of 4-ATP at 1078 cm^−1^. A high correlation between the 4-ATP Raman intensity and concentration is indicated (R^2^ = 0.996).

In addition, Table 1 compares the results of the 4-ATP detection by different core–shell nanoparticles’ SERS. In general, precious metals of different shapes such as Ag and Au, non-metallic oxides such as TiO_2_ and SiO_2_, and polymer PDMS are used as the SERS’s active base to detect 4-ATP. The limit of detection (LOD) of these substrates is not less than 10^−10^ mol/L, while the introduction of metal backbones can be up to 10^−12^ mol/L. The LOD was calculated according to the following formula: LOD = 3σ/k. The SERS spectrum of the blank substrate was repeated 10 times, and the standard deviation of the band intensity was calculated, which was σ. k was the slope of the standard curve The Au@AgNPs/PDMS substrate prepared in this study has a high sensitivity, and the LOD of 4-ATP is as low as 0.28 × 10^−11^ mol/L. Simultaneously, the substrate preparation process is simpler and faster than that of the metal skeleton, which has better commercial application prospects.

### 2.6. Determination of Dicofol with the Au@AgNPs/PDMS SERS Substrate

Figure S2c shows the Raman spectra of the dicofol samples on tinfoil and PDMS film, and the difference between the two can be ignored. There are few related studies on dicofol, so the reference materials are limited. Density-functional theory (DFT) was applied to calculate the standard Raman spectra of dicofol to confirm the theoretical distribution of the Raman peaks and to verify the accuracy of the experimental spectra [45]. The molecular configuration of dicofol was constructed using Gauss View 6.0. The theoretical Raman spectra of dicofol were calculated using the B3LYP/6-311 G (D, P) group of the B3LYP method of DFT. The calculation results of the dicofol Raman frequency were checked on the Gauss View 6.0 software platform, and the corresponding correction factors were used for correction. The standard Raman spectrum of dicofol calculated by DFT is shown in Figure S2c. The spectral curves of eight gradient concentrations of dicofol are shown in Figure 3c. The higher the content of dicofol is, the higher the Raman intensity of the tea sample is. The Raman spectra of dicofol on tin foil and Au@AgNPs/PDMS show that Raman peaks exist at 489, 888, 1055, 1094, and 1463 cm^−1^ and 489, 619, 709, and 1261cm^−1^, respectively. The corresponding vibration forms are explained in Table S1. The standard curve was constructed with the band intensity at 489cm^−1^. Figure 3d shows that the coefficient of determination of the dicofol content and Raman intensity is 0.9791. The LOD of dicofol on the Au@AgNPs/PDMS substrate prepared in this study was 0.32 ng/kg.

Environmental fluctuations and tea sample complexity have a certain impact on spectral intensity, so the spectral data are processed by stoichiometry to improve the model reproducibility and prediction accuracy. Figure 3e shows all the raw Raman spectra of dicofol under eight concentration gradients (15 spectra per collected gradient). Figure 3f shows the PLS modeling results of the original Raman spectrum. On this basis, five algorithms (SG, SNV, MSC, first derivative, and second derivative) were used to preprocess the Raman spectral data. The spectral shifts were in the range of 300–2000 cm^−1^, and each spectrum included 667 variables.

Table 2 shows the results of the different pretreatment models of dicofol. SNV and MSC show poor modeling results. The noise removal ability of these two preprocessing algorithms is not well-demonstrated in this experiment. The derivative treatment did not significantly optimize the original spectra or even slightly reduce them. After smoothing, the PLS modeling results were the best (Rp = 0.9906, and RMSECP = 4.8120), and the residual predictive deviation (RPD) value was 7.2341, indicating that the prediction ability of the SG-PLS model was better. Therefore, the subsequent variable selection algorithm was based on the SG smoothing preprocessing.

### 2.7. Variable Selection and Models Development

After data preprocessing, we carried out variable selection to improve the prediction accuracy of the model. In this study, four variable selection algorithms (CARS-PLS, Si-PLS, SPA-PLS, and UVE-PLS) were used to process the data.

#### 2.7.1. CARS-PLS Model

**Figure 4:**
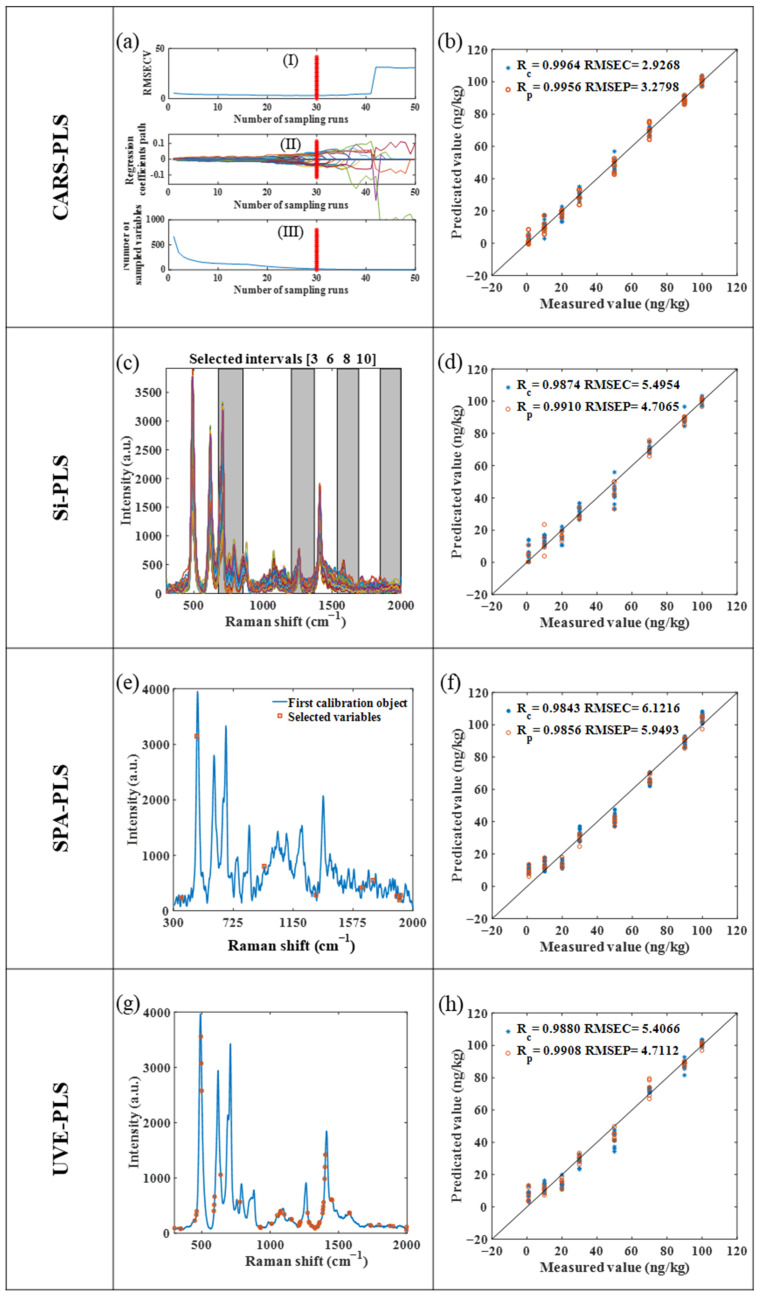
Four variable-filtering graphs and PLS modeling results of dicofol spectral data. (**a**,**b**) Variable-filtering and modeling results of CARS-PLS model; (**c**,**d**) variable-filtering and modeling results of Si-PLS model; (**e**,**f**) variable-filtering and modeling results of SPA-PLS model; (**g**,**h**) variable-filtering and modeling results of UVE-PLS model.

After the SG smoothing of the raw spectral data, the CARS-PLS algorithm was used for spectral variable selection. Figure 4a(I) shows the periodic variation rule of RMSECV, which reaches the minimum value of 3.008 in the 30th cycle. After removing the irrelevant variables from the model, the RMSECV value gradually increases. Figure 4a(II) shows the periodic variation rule of the regression coefficient. The regression coefficient values for the first 15 cycles were close to zero. After that, the regression coefficient of the strong competition variable increases, while that of the weak competition variable decreases. Figure 4a(III) shows the trend of the number of variables, which decreases faster in the first six periods and gradually slows down afterward. This change indicates that the refinement of the variable selection is realized by quickly eliminating any redundant variables, which are reduced from 667 variables to 21. The variable-selecting and modeling results of the CARS-PLS model are shown in Figure 4a,b.

#### 2.7.2. Si-PLS Model

The model construction process of Si-PLS is as follows. The spectral data are divided into several equidistant intervals, and then the correlation information of the interval variables and analytes is recombined to establish a stable prediction model. In this study, the entire spectrum is divided into 10 subintervals. By recombining the 2nd, 3rd, 4th, and 10th subintervals, the optimal PLS model is obtained (Rp = 0.9910), which is reduced from 667 variables to 267. The variable-selecting and modeling results of the Si-PLS model are shown in Figure 4c,d.

#### 2.7.3. SPA-PLS Model

SPA randomly selects a variable in the data matrix, calculates the projection of the random variable on the remaining variables, and selects the variable with the least-redundant information content to improve the modeling effect by projecting the information. The variable selection results of SPA are shown in Figure 4e, nine characteristic variables are selected, and finally the prediction model of dicofol in tea is established. Compared with the model prediction accuracy of PLS, that of SPA-PLS is reduced, and the modeling results are shown in Figure 4f.

#### 2.7.4. UVE-PLS Model

The UVE algorithm eliminates invalid variables by adding artificial noise variables to the sample variables and cross-verification based on PLS regression coefficients, thus improving the modeling effect. The maximum modeling principal component number is 20, and the artificial noise variable is 160. Figure 4g shows the UVE variable selection plot for dicofol, which is reduced from 667 variables to 45. The modeling results of the UVE-PLS model are shown in Figure 4h.

### 2.8. Models Effect Evaluation

Table S2 clearly shows the modeling results of the four variable selection algorithms. The RPD value is used to evaluate the prediction ability of the four models, and the RPD value is always greater than 4. CARS-PLS achieves the best prediction (Rp = 0.9964, and RMSEP = 2.9268), followed by Si-PLS, UVE-PLS, and PLS, while SPA-PLS has the worst prediction. The number of variables is reduced from 667 to 21, and the model is obviously simplified. The CARS-PLS prediction effect is the best, which proves that the variable selection algorithm can eliminate redundant variables and improve the modeling effect.

## 3. Discussion

The residue of dicofol in tea leaves is harmful to human health. A method for detecting dicofol in tea by combining stoichiometry with surface-enhanced Raman spectroscopy (SERS) technology was proposed in this study. The core–shell structure can combine the advantages of the two metals, while the PDMS film has good flexibility and strong corrosion resistance. Therefore, we prepared core–shell Au@AgNPs with excellent enhancement properties, high stability, and high sensitivity. Then, core–shell nanoparticles were attached to the surface of the PDMS by physical deposition, and the Au@AgNPs/PDMS substrate was prepared.

A Au@AgNPs/PDMS substrate has excellent stability and reproducibility and can detect a 10^−11^ mol/L 4-ATP signal, showing high sensitivity. This may be due to the high enrichment of the core–shell Au@AgNPs on the PDMS membranes resulting in more SERS “hot spots”. This substrate was used for the SERS detection of dicofol in tea, and a standard curve was established. R^2^ = 0.9791, indicating a good correlation between the Raman intensity and sample concentration.

Considering the influence of noise and sample complexity on spectral intensity, the spectral data were processed by chemometrics. The algorithm modeling effect of SG smoothing combined with CARS-PLS was the best (Rp = 0.9964, RMSEP = 2.9268, and RPD = 10.6145). The use of chemometric algorithms effectively removed the influence of redundant information and improved the modeling effect.

In summary, the highly efficient and sensitive SERS detection method for dicofol in tea proposed in this study had good repeatability and was easy to operate. Therefore, it has broad application prospects in medicine, food safety, the life sciences, and other fields.

## 4. Materials and Methods

### 4.1. Chemicals Reagents and Materials

The gold nanoparticles were prepared from chloroauric acid (AuCl_3_·HCl·4H_2_O) and trisodium citrate solution. Silver nitrate (AgNO_3_), ascorbic acid (AA), and prepared gold nanoparticles were used to synthesize gold and silver core–shell nonsolutions. Polydimethylsiloxane (PDMS) films were retreated with Piranha solution (H_2_SO_4_:HNO_3_ = 3:7 (*v*/*v*)) and APTES solution (purchased from Zhongke Experimental Materials Co., Ltd., Hefei, China). 4-ATP is a signaling molecule. Dissolve dicofol in an ethanol solution. Reagents used are of analytical grade purity and are intended for direct use unless otherwise stated. Wash each glassware with ultra-pure water before usage. Green tea was purchased from a supermarket in Jingkou District, Zhenjiang City.

### 4.2. Instrumentation

The substances used in the experiment were weighed using a microanalytical balance (Sartorlus, Shanghai, China), and the green tea powder sample was digested with a microwave digester (MARS 6) (CEM, Matthews, NC, USA). AuNPs and core–shell Au@AgNPs were characterized with ultraviolet spectrophotometer (UV–Vis) (Agilent Technologies Inc., Santa Clara, CA, USA), laser particle size analyzer (Particle Size Analyzer, Malvern, UK), scanning electron microscope (SEM) (JEOL Ltd., Tokyo, Japan), and JEM-2100 HR transmission electron microscopy (TEM) (JEOL Ltd., Japan). Spectral data of 4-ATP and dicofol were collected and processed by confocal micro-Raman spectroscopy (XploRA PLUS, HORIBA, Palaiseau, France) The excitation wavelength was 638 nm, and the objective lens was 10×. The laser power was 400 mW, the cumulative number of sample collection was 1, and the collection time was 1s. MATLAB 2016b (MathWorks, Natick, MA, USA) was used to process the spectral data. Gauss View 6.0 (Gaussian Inc. Chicago, IL, USA) was used to construct the molecular configuration of dicofol.

### 4.3. Synthesis of Flexible Substrates for Au@AgNPs/PDMS

First, the gold nano solution was prepared by seed growth method: take 30 mL of ultrapure water in a 100 mL beaker, add 425 μL of HAuCl_4_ (5 g/L) solution, heat to boiling state, hold for 2 min under the condition of magnetic stirring at 1200 rpm, then add 150, 200, 250, 300, 350, and 400 μL of trisodium citrate solution with a mass fraction of 1%, and continue to heat until the color of the liquid no longer changes. Allow the liquid to cool to room temperature and store in a refrigerator at 4 °C. At this point, gold nanoparticle solutions of different diameters were prepared. Gold nanoparticles (34 nm) with the best SERS-enhancement effect were selected for subsequent experiments.

Then the preparation of gold and silver core–shell nanoparticles was carried out: 6 mL of optimized gold nanoparticle solution (34 nm) was taken in a test tube, and 30, 45, 60, 75, 90, and 105 μL of ascorbic acid solution with a concentration of 10 mmoL/L were added after 10 min of ultrasonication. After shaking at 1000 rpm for 2 min, add the same concentration of AgNO_3_ solution. Refrigerate at 4 °C after 7 min of continuous shaking.

After that, the amination functionalization of the PDMS membrane was carried out. Piranha solution (H_2_SO_4_:HNO_3_ = 3:7 (*v*/*v*)) was added to the PDMS film. Shake for 10–30 s, take out the PDMS film, clean with ultrapure water, blow dry with high-purity nitrogen, repeat this step 3~4 times, and then put it in a clean surface dish. Hydroxylated PDMS films were soaked in APTES ethanol solution (5%, *v*/*v*) for 3 h (70 °C). Amine-functionalized PDMS film was obtained after washing with ultra-pure water and drying.

Finally, the core–shell Au@AgNPs were deposited in aminated PDMS by physical method to form Au@AgNPs/PDMS film.

### 4.4. Feasible Characterization of the Composite Raman Substrate

In order to determine whether the substrate material would affect the subsequent detection of substances, the Raman spectra of blank tin foil, blank PDMS, and blank Au@AgNPs/PDMS substrates were collected. The original Raman spectra of 0.1 mol/L dicofol standard solution on tin foil and on blank PDMS film were collected. In order to prove that the SERS substrate had strong SERS-enhanced effect, 10^−6^ mol/L Raman signaling molecule 4-ATP was added into dicofol solution. The Raman spectra of the mixed solution on the tin foil and SERS substrate were collected, and the changes in Raman spectral intensity before and after using the enhanced base were compared.

### 4.5. Sample Preparation

A bag of ordinary green tea was purchased from Jiangsu University supermarket. The powdered green tea samples were stored in the refrigerator at 4 °C for the subsequent experimental operation. First, 0.200 g tea powder was added with 9 mL concentrated HNO_3_ and different concentrations of dicofol standard solution, standing for digestion for 12 h. The tea samples were digested according to the general operating rules of microwave digestion instrument. The digestion conditions were set as follows: the digestion temperature was 190 °C, the heating time as 20 min, and the constant temperature time was 10 min. After digestion, the digestion tank should be taken out after cooling to room temperature. The digestion solution was clear and transparent without precipitation [20]. The digestion solution was filtered by 0.22 μm membrane and stored at 4 °C.

### 4.6. SERS Detection of 4-ATP and Dicofol

SERS spectra of experimental samples were acquired using a confocal Raman microscopy system, and spectral data were processed using LabSpec6 software (Lab Spec_6_5_2) inside the system. The excitation power used in the experiment was 400 mW, the cumulative number of sample acquisitions was 1, and the acquisition time was 1 s. All Raman spectra were obtained under a 10× objective with an excitation wavelength of 638 nm. Using 4-ATP as the signaling molecule, the enhanced effects of the prepared gold nanoparticles, core–shell Au@AgNPs, and Au@AgNPs/PDMS were investigated.

To analyze the SERS-enhanced effect of gold nanoparticles of different sizes, 10 μL of 4-ATP (0.1 mmol/L) solution was mixed with 990 μL of AuNPs. After 10 min, take 5 μL of the mixed liquid for Raman detection. In order to explore the effect of silver-shell thickness on the SERS performance of core–shell Au@AgNPs, 10 μL of 4-ATP (0.1 mmol/L) solution was mixed with 990 μL of core–shell Au@AgNPs. After 10 min, take 5 μL of the mixed liquid for Raman detection. Raman spectra of 4-ATP in six AuNPs with different particle sizes and six core–shell Au@AgNPs with different silver-shell thicknesses were collected.

An Au@AgNPs/PDMS substrate with a size of 6 mm × 6 mm was placed on tin foil for SERS testing. To investigate the sensitivity of the Au@AgNPs/PDMS substrate, different concentrations of 4-ATP solution (10^−6^, 10^−7^, 10^−8^, 10^−9^, 10^−10^, 10^−11^, and 10^−12^ mol/L) were added to the substrate for SERS detection. To investigate the reproducibility of the Au@AgNPs/PDMS substrate, 10 μL of 4-ATP (0.1 mmol/L) solution was dropped on the substrate, and 15 points were uniformly collected in mapping mode in a region of 3 mm × 2 mm, and the standard deviation was calculated. To explore the stability of the Au@AgNPs/PDMS substrates, three substrates were stored in closed Petri dishes, and Raman measurements were performed dropwise with 5 μL of 4-ATP (0.1 mmol/L) solution at 10, 20, and 30 days. Each substrate was uniformly collected from 25 points in the mapping mode in a region of 3 mm × 3 mm, and the intensity of the 25 points was averaged. Finally, the standard deviation of the three substrates was calculated.

The SERS spectra of dicofol standard solution on tin foil were collected, and the SERS spectra of eight gradient concentrations (1, 10, 20, 30, 50, 70, 90, and 100 ng/kg) spiked tea samples on the Au@AgNPs/PDMS substrate were collected. The calculation method of dicofol content in tea samples is described in the supplementary materials.

Due to the complexity of tea samples and the variability of the external environment, the acquired spectra were affected by fluorescence interference and had a certain degree of baseline drift. Therefore, it was necessary to perform baseline processing and data smoothing on the acquired spectra to improve the spectral quality. The de-baseline parameters selected for this study were set as follows: background fit was polynomial, degree = 7, and maximum points = 256. In addition, control the data smoothing effect by changing the function type and parameters [46]. The function type selected for this study was Denoise, and the parameters were set as follows: degree = 1, and size = 1.

### 4.7. Data Preprocessing and Models Development

#### 4.7.1. Spectra Preprocessing

To minimize baseline drift and interference of redundant background information, Raman spectra were preprocessed by Savitzky–Golay (SG), spectral multivariate scattering correction (MSC), variable normalization (SNV), first derivative, and second derivative. These preprocessing methods can better remove the noisy data in the spectrum and eliminate the baseline drift, which is significant to improve the prediction effect and generalization ability of the model. In this study, five methods were selected to preprocess the original Raman spectral data of dicofol and establish PLS model.

#### 4.7.2. Variable Selection

Multivariate calibration methods can improve spectral modeling by selecting spectral variables. In this study, four variable selection algorithms were used to process the spectrum data of dicofol by synergy interval partial least squares method (SI-PLS), uninformed variable elimination algorithm (UVE-PLS), successive projection algorithm (SPA-PLS), and competitive adaptive reweighted sampling (CARS-PLS). SI decomposes all variables into several equidistant subintervals and then combines 1, 2, or more subintervals to construct the model. The joint interval can compensate for the lack of information of a single interval and improve the accuracy of the model [47,48]. The UVE algorithm is based on PLS and can eliminate uninformative variables from the data [49]. SPA is a forward characteristic variable selection method, which can select the combination of variables with the least-redundant information content and least collinearity [50]. CARS treats each variable as an independent individual through adaptive weighted sampling and retains the variable with a larger weight as a new subset, which can effectively remove irrelevant variables and reduce the influence of collinear variables [51,52].

#### 4.7.3. Model Assessment

Five pretreatment algorithms based on PLS model and four variable selection algorithms were combined to establish the best prediction model for dicofol concentration. The 120 spectral data (667 independent variables) were divided into 72 calibration sets and 48 test sets. The calibration set was used to build the partial least squares model, and the test set was used to verify the generalization ability of the evaluation model. The model was evaluated by correlation coefficient of calibration (Rc), root mean square error of calibration (RMSEC), correlation coefficient of prediction (Rp), and root mean square error of prediction (RMSEP). The smaller the RMSEC and RMSEP values were, the closer R was to 1, and the more accurate and stable the model was. Algorithmic processing of all SERS data took place in MATLAB 2016b.

## 5. Conclusions

This study proposed a method for detecting dicofol in tea by combining stoichiometry with SERS technology. The core–shell Au@AgNPs were prepared using ascorbic acid as the reducing agent and then attached to the aminationized PDMS membrane using physical deposition methods to obtain a Au@AgNPs/PDMS substrate. Under the condition of an excitation wavelength of 638 nm, the Raman spectra of the 4-ATP and dicofol gradient concentrations on the SERS substrate were collected, and a linear relationship between the spectral intensity and gradient concentration was established. The LOD for 4-ATP was 0.28 × 10^−11^ mol/L for this substrate and 0.32 ng/kg for dicofol in tea, which is much lower than the safe limit of dicofol in tea (200 ng/kg). The modeling effects of five preprocessing algorithms and four variable selection algorithms based on PLS model were compared. The algorithm combining SG and CARS-PLS modeled the dicofol data the best (Rp = 0.9964, and RPD = 10.6145). Combined with stoichiometry, SERS technology can be used for the efficient and sensitive detection of dicofol in tea.

## Figures and Tables

**Table 1:** Comparison of the results of detecting 4-ATP using different SERS bases.

SERS Substrate	Detection Level (mol/L)	Reference
Al-TiO_2_-ZIF-8-Ag	10^−9^	[38]
Au-rice	10^−6^	[39]
IP6@Au NPs	10^−7^	[40]
CFP@PDA@AuNPs	10^−9^	[32]
Ag@Au NWs	10^−9^	[41]
g-C_3_N_4_/Au NWs	10^−8^	[42]
PDMS/TiO_2_/Ag	10^−9^	[29]
Au-SiO_2_ IO film	10^−10^	[35]
Ag/rGO	10^−10^	[43]
FP/Ag/ZIF-8	10^−12^	[44]
Au@AgNPs/PDMS	10^−11^	This study

**Table 2:** Modeling results of five pretreatment algorithms for dicofol.

Model	Calibration SET		Prediction Set		
	Rc	RMSEC	Rp	RMSEP	RPD
SG-PLS	0.9869	5.6093	0.9906	4.8124	7.2341
MSC-PLS	0.8714	17.3319	0.8690	17.7664	1.9595
SNV-PLS	0.8737	17.1274	0.8734	17.5182	1.9872
1st-PLS	0.9866	5.7014	0.9906	4.8204	7.2221
2st-PLS	0.9861	5.8090	0.9893	5.1056	6.8187

## Data Availability

The data presented in this study are available on request from the corresponding authors.

## Supplementary Materials

The following supporting information can be downloaded at https://www.mdpi.com/article/10.3390/molecules28145291/s1. Figure S1: TEM images of AuNPs and core–shell Au@AgNPs; Figure S2: (a) Raman spectra of a blank PDMS film and a blank Au@AgNPs/PDMS substrate, (b) Raman spectra of blank tin foil, 4-ATP (10^−6^ mol/L) on tin foil and 4-ATP (10^−6^ mol/L) on Au@AgNPs/PDMS substrate, (c) standard Raman spectrum calculated by DFT of dicofol and Raman spectra of 0.1 mol/L dicofol standard solution on tin foil and on a blank PDMS film; Table S1: Vibrational wavenumbers and assignments of dicofol; Table S2: Statistical performance results of dicofol detection by PLS model based on four different variable selection methods.

## Sample Availability

Samples of the compounds are available from the authors.
