# Supplementary material for: Determination of Dicofol in Tea Using Surface-Enhanced Raman Spectroscopy Coupled Chemometrics
Source: Molecules. 2023 Jul 8;28(14):5291. doi: 10.3390/molecules28145291 (PMC10386380; doi:10.3390/molecules28145291)
Supplement: Supplementary file 1 [file molecules-28-05291-s001.zip › molecules-2450334-supplementary.pdf]

Supplementary Materials

Determination of dicofol in tea using surface-enhanced Raman spectroscopy coupled chemometrics

Qian Ke [1], Limei Yin [1,2], Heera Jayan [1], Hesham R. El-Seedi [3,4], Paula L. Gómez [5], Stella M. Alzamora [5], Xiaobo Zou [2,5] and Zhiming Guo [1,6]*

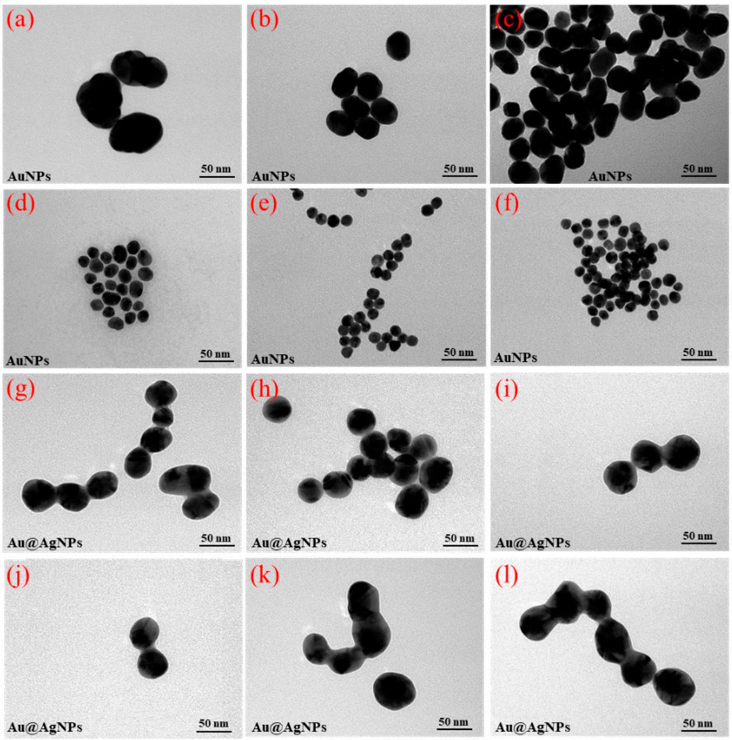

Figure S1. TEM images of AuNPs and core-shell Au@AgNPs. AuNPs are obtained by adding different volumes of 1% trisodium citrate (150, 200, 250, 300, 350, 400μL) to 425μL of $AuCl_3{\cdot}HCl{\cdot}4H_2O$ (5 g/L); core-shell Au@AgNPs is obtained by adding different volumes of equal amounts of $AgNO_3$ and ascorbic acid (30, 45, 60, 75, 90, 105μL) to 6 ml of AuNPs (34 nm)

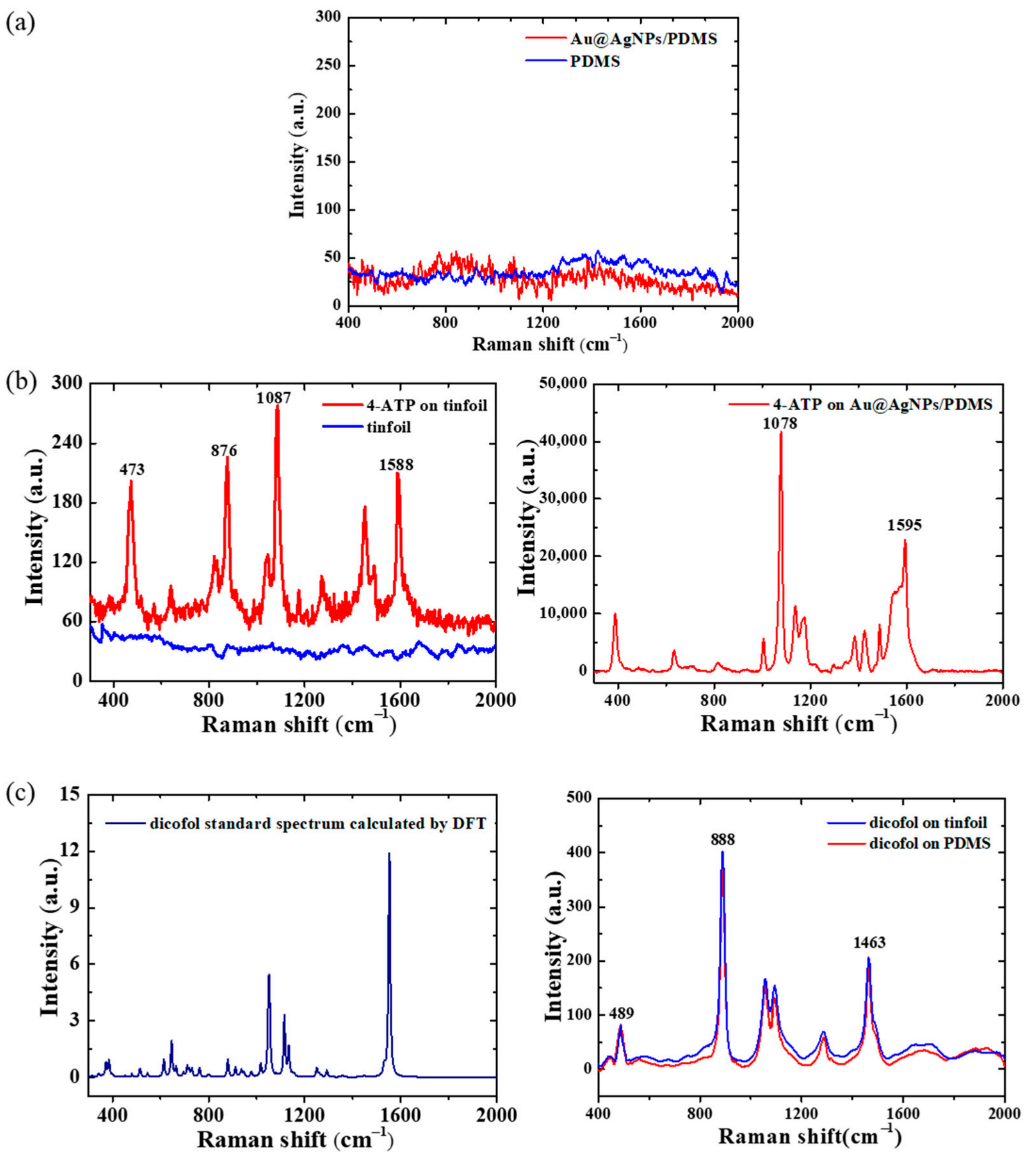

Figure S2. (a) Raman spectra of a blank PDMS film and a blank Au@AgNPs/PDMS substrate (b) Raman spectra of blank tin foil, 4-ATP (10^{-6} mol/L) on tin foil and 4-ATP (10^{-6} mol/L) on Au@AgNPs/PDMS substrate (c) standard Raman spectrum calculated by DFT of dicofol and Raman spectra of 0.1 mol/L dicofol standard solution on tin foil and on a blank PDMS film

Table S1. Vibrational wavenumbers and assignments of dicofol

Experiment shift (cm^{-1})	Calculated shift (cm^{-1})	Assignments
489	481	C-C out of plane rock
619	614	C-H in plane wag
709	710	C-C out of plane rock
888	880	C-Cl out of plane wag/ C-C out of plane rock
1055	1053	C-Cl stretch/ C-C stretch
1094	1091	C-C stretch /C-H in plane wag
1261	1260	C-C stretch/ C-H in plane wag
1463	1447	C-C stretch/ C-H in plane wag

Table S2. Statistical performance results of dicofol detection by PLS model based on four different variable selection methods

Model	Calibration set		Prediction set		
	Rc	RMSEC	Rp	RMSEP	RPD
CARS-PLS	0.9964	2.9268	0.9956	3.2798	10.61452
Si-PLS	0.9874	5.4954	0.9910	4.7065	7.396898
SPA-PLS	0.9843	6.1216	0.9856	5.9493	5.851697
UVE-PLS	0.9880	5.4066	0.9908	4.7112	7.389519

The content of dicofol in the sample was calculated according to Formula (S1)

$$X = \frac{\rho \times V}{m} \times 10^{-6} \quad \text{(S1)}$$

X the content of dicofol in the sample (ng/kg);

ρ the concentration of dicofol in the sample solution calculated from the standard curve (μg/mL);

V final volume (mL)

m sample weight (g)
